# Assessing physical activity promotion in different settings and how its associated with public participation during COVID-19 epidemic: evidence from national policy evaluation

**DOI:** 10.1186/s12889-023-16690-9

**Published:** 2023-09-12

**Authors:** Narakorn Wongsingha, Dyah Anantalia Widyastari, Burathep Chokthananukoon, Niramon Rasri, Piyawat Katewongsa

**Affiliations:** 1 Thailand Physical Activity Knowledge Development Centre (TPAK), Salaya, Phutthamonthon, Nakhon Pathom, 73170 Thailand; 2 Institute for Population and Social Research, Mahidol University, Salaya, Phutthamonthon, Nakhon Pathom, 73170 Thailand; 3 Thai Health Promotion Foundation, Thung Maha Mek, Sathorn, Bangkok, 10120 Thailand

**Keywords:** Policy evaluation, Investments, National PA guidelines, Health promotion, Well-being

## Abstract

**Background:**

Various interventions, programs and policies have been implemented to improve physical activity (PA) levels worldwide. However, countries continue to face barriers and challenges in achieving their targets. To date, there is a lack of study on the evaluation of physical activity (PA) promotion and how it’s associated with public participation.

**Methods:**

This study assessed PA promotion in eight different settings in terms of policy availability, policy implementation, and public participation in PA programs. Policy availability was assessed by reviewing 384 policy and strategy documents, rules, regulations, legislation, and guidelines on PA. We scored the documents by using the Comprehensive Analysis of Policy on Physical Activity (CAPPA) framework. Data to assess policy implementation and public participation were taken from the Thailand Report Card Survey 2021 (TRC2021), and the Thailand Surveillance on Physical Activity (SPA) 2021. Both surveys comprised over 5,000 nationally-representative samples from on-screen, face-to-face interviews, and an online self-administered survey. We scored the policy implementation and public participation based on respondents’ response towards policy implementation and participation indicators. A grading scheme was applied to indicate how successful an investment has been made.

**Results:**

Public education and mass media received the highest average score in policy availability, implementation and public participation in PA program (67.9%, grade B), followed by active urban design (66.1%, grade B-) and active transport (63.7%, grade B-). Workplace, whole-of-school, and community-wide initiatives were the investments with the lowest scores, implying low availability, limited implementation, and less accessibility to public. Females were less likely to participate in active transport, active urban design, sports/recreation for all, workplace activity, and community-wide initiatives. Age and educational attainment were consistent predictors of utilization in all investments.

**Conclusions:**

With varying degrees of policy availability and accessibility, public participation in PA investments is likely to be constrained by biological and socioeconomic inequality. Future investments should aim at providing generalized or tailored interventions to ensure equal access and participation for all segments of the population.

**Supplementary Information:**

The online version contains supplementary material available at 10.1186/s12889-023-16690-9.

## Introduction

Studies have documented growing physical inactivity worldwide. Globally, 28% of adults and 81% of adolescents were insufficiently physically active in 2016 [1, 2], with a slightly higher proportion among females. A significant reduction in the cumulative minutes and the proportion of population with sufficient moderate-to-vigorous-physical activity (MVPA) was reported during the COVID-19 pandemic, largely as a result of government mandates to contain the virus e.g., lockdown, closures of public facilities and non-essential business, and the shift from onsite to remote learning/working [3–6]. Physical activity (PA) inequality was also more profound during the pandemic, as indicated by the lowest level of PA occurring among disadvantaged groups of the population, i.e., those with no income, the unemployed, who have no access to PA facilities, persons age 60 + years, and low income individuals, [7]. While the Covid pandemic has gradually subsided, physical inactivity remains a great problem worldwide. Physical inactivity is responsible for 7.2% of all-cause mortality and 7.6% of cardiovascular disease death, and this burden is doubled in middle- and low-income countries [8]. Not only does this burden the global health care system (by an estimated $54 billion annually), physical inactivity also economically burdens families, as households had to pay nearly $10 billion for associated costs of physical inactive in 2013 [9].

Various interventions, programs, and policies have been implemented in different settings to improve PA level. The International Society for Physical Activity and Health (ISPAH) has proposed a package of investment strategies to promote PA known as “*Eight investments that work for physical activity*” [10]. The eight investments for PA promotion include: (1) Whole-of-school programs; (2) Active travel/transport; (3) Active urban design; (4) Healthcare service; (5) Public education/mass media; (6) Sports and recreation for all; (7) PA promotion in the workplace; and (8) community-wide initiatives [10]. The 8-investments package also align with the Global Action Plan on Physical Activity (GAPPA) as the World Health Organization (WHO)’s blueprint strategy that consists of (1) Creating a social environment which values PA (‘Active Society’); (2) Creating an environment conducive to PA (‘Active Environment’); (3) Creating opportunities for PA (‘Active People’); and (4) Creating systems that facilitate PA (‘Active Systems’) [11].

While the recommendation on ‘8-investments that works for PA’ were driven from empirical evidence and GAPPA as guidelines to improve PA of the population, to date, there has been no study reported on how to assess or evaluate the implementation of policies/investments corresponding with the two strategies as a comprehensive analysis. There is also a lack of evidence pertaining to the coverage of each investment and the causes of gaps in the policy planning/development. The existing studies mostly focused on research into the effectiveness of the interventions (investments), i.e., the effect of the whole-of-school program in improving PA of the student population [12–14]; or how active urban design and active transportation investments may help increase PA opportunity [15, 16]. There have been economic analyses of particular investments for health, e.g., health economic impact of active transport [17, 18], cost-effectiveness of mass media [19], and health-care based [20] or community-based interventions [21, 22] in improving PA. However, there have been few studies which evaluated the underlying policy itself. Moreover, of the few policy analyses/evaluations that have been conducted, most focused only on one investment. For instance, A Bauman, BJ Smith, EW Maibach and B Reger-Nash [23] evaluated mass media campaigns in the USA, while KR Allison, K Vu-Nguyen, B Ng, N Schoueri-Mychasiw, JJ Dwyer, H Manson, E Hobin, S Manske and J Robertson [24] evaluated Daily Physical Activity (DPA) policy implementation in Canada, and E Holt, T Bartee and K Heelan [25] analyzed the policy to integrate PA into the school curriculum.

This study assessed the implementation of PA promotion in different settings following the 8-investments recommended by ISPAH. More specifically, we assessed the policy implementation (availability, and how far a policy/investment has been implemented), and also public participation in PA programs. We developed a grading system to facilitate comparison of each population group by access to and benefit from the implementation of different PA policies across the 8-investment areas. The analysis categorizes policy domain following the GAPPA framework to ensure all strategies in PA promotion are covered. The results from the study should be beneficial for countries, particularly policymakers and government, in planning and assessing the impact of PA promotion/policy implementation. This study also provides guidelines for countries to align their PA promotion investments with global targets and indicators. Further, the results of this study should add to the body of knowledge in the policy evaluation field by demonstrating a new method of analysis involving WHO’s GAPPA and ISPAH’s 8-investments for PA.

## Methods

This study is a policy evaluation of PA promotion in eight different settings, based on ISPAH’s recommendation of strategic investments. We assessed the PA promotion strategies in three dimensions: (1) Policy availability; (2) Status of implementation; and (3) Public participation in PA programs.

### Policy availability

Desk review was performed to assess policy availability. In the first stage, we collected 384 policy and strategy documents, rules, regulations, legislation, and guidelines on PA from online public databases. The documents then were divided into four domains of GAPPA – Global Action Plan on Physical Activity [11]: (1) Active Society policies; (2) Active Environment policies; (3) Active People policies; and (4) Active System policies. In the second stage, three experts evaluated and scored the policy documents by using a matrix adopting the Comprehensive Analysis of Policy on Physical Activity (CAPPA) framework [26]. We excluded policies (*n* = 47) of which the three experts failed to reach agreement on the scoring. We employed three indicators *Type of policy, Stage of policy cycle, and Policy resources* and defined each category following Pogrmilovic, et.al [26]. We categorized the *types of policy* into 1) Formal written – if the documents comprise of strategies, plans, or regulations that have been officially enacted and/or endorsed; 2) Unwritten formal – official statements made in public by an official that were not documented in formal writing (.e.g., statement of a Senator published in a newspaper); 3) Written standard/ guidelines– a set of guide choices, recommendation to perform certain behaviors, practices, or processes but do not create an obligation for stakeholder adherence (e.g., national guidelines on PA); 4) Formal procedures – formal actions conducted or authorized by an official body that are indicative of commitment regarding PA (e.g., documents on surveillance in PA); 5) Informal policies – include norms, actions, voluntary codes of practice supported by an official body that are indicative of the body’s commitment regarding PA (e.g., no fine for cyclist who ride a bike in the footpath in the area where bike lane is unavailable as an indication of supporting active transport); or 9) Unclear – if the policy falls outside the 8 categories. We scored category 1–4 as 1, and the rest as 0. We categorized the *stages of policy cycle* into 1) Agenda setting, 2) Formulation, 3) Endorsement/legitimization, 4) Implementation, 5) Evaluation, 6) Maintenance, 7) Termination, and 8) Succession. Category 4–6 were scored as 1, whereas the rest as 0. *Policy resource allocation* was categorized into 1) Management resources, and 2) Budget resources. Score 1 was assigned to policies with both resources available, otherwise was scored 0. In the final stage, we counted the number of policies that met the CAPPA criteria in each domain, assigned an equal weight (0–3), and calculated the final score (expressed as a percentage) for policy availability (Supplementary materials).

### Policy implementation

Data for evaluating policy implementation was derived from the Thailand Report Card Survey 2021 (TRC2021), and the Thailand Surveillance on Physical Activity (SPA) 2021. Both surveys were conducted by the authors’ institute. TRC2021 was employed to analyze policy implementation and accessibility in the domain of whole-of-school programs for Thai youth age 5–17 years. This survey involved nationally-representative samples of 6,078 students from 121 schools, disaggregated by region, residential area, school size, gender, and age group. To comply with COVID-19 prevention measures, data was collected by using on-screen, face-to-face interviews where the interviewer, respondents and their guardian interacted in real time mediated by screen media (e.g., laptop, tablet or smartphone). SPA2021 was used to assess policy accessibility and implementation on the rest of PA promotion in 7 domains. A total of 7,847 nationally-representative samples from all 77 provinces in Thailand were included in the analysis. The data was collected as an online survey where the respondents – and their guardian if the respondents were children – entered their responses into a LimeSurvey web application.

We measured policy implementation as the percentage of policies on PA being implemented either at the national or community level, based on the perspective of the beneficiaries: Thai children and adolescents (aged 5–17 years, *N* = 6,078) for the whole-of-school domain; working aged Thais (aged 20–59 years, *N* = 5,895) for the workplace domain; and the general public (Thais aged 5 years and over, *N* = 7,847) for the rest of the domains. The questions to assess policy implementation in each domain of the 8-investments are shown in Table 1. We calculated the percentage of policy implementation from every ‘yes’ answer (scored 1) from the respondents in each domain divided by the total number of beneficiaries.

**Table 1:** Indicators to assess policy implementation and public participation in 8-investments

Domain	Investments	Policy implementation indicators	Public participation indicators
1	Whole-of-school approach	1) Does this school have PA promotion policies? 2) How often (frequency and duration) is physical education (PE) administered for each grade? 3) Is PE taught by teachers who have completed Physical Education or Sports Science education? 4) In which period of the day does the school encourage PA?	Do you (students) move moderate or vigorously, whether it's playing, exercising, or engaging in sports, doing housework, or helping parents with work, including walking or cycling for at least 60 min a day?
2	Active transport	Is there a pathway for walking, jogging, running or biking in the community?	Have you ever used the pathway for walking, running, or cycling in the community area for commuting or traveling between places?
3	Active urban design	Is there any area in your community that is designed to facilitate PA (exercise, play, and recreation)?	Have you ever used the community area for PA, exercise, play, and/or recreation?
4	Healthcare	To your knowledge, is there any healthcare facility in your locality that provides advice on PA?	Have you received any advice about PA or exercise provided by the healthcare provider?
5	Public education and mass media	Have you ever heard of any campaign on PA facilities and programs? If yes, through which channels?	Have you been exposed to any campaign on PA through various media channels? If yes, what information have you received from the campaign?
6	Sports/recreation for all	Do you know if there is any organized sports or PA for persons of all ages available in your community?	Have you participated in any exercise, sports, and/or virtual/online events designed for all ages?
7	Workplace	Are there in-house PA campaigns, events, and competitions, virtual/online activities, and a supportive environment for PA, e.g., which promotes the use of stairs instead of elevators and reduces sedentary behavior in the workplace?	Have you participated in PA, playing sports, or virtual/online events organized by your workplace to be physically active and reduce sedentary behavior?
8	Community-wide initiative	Has there been any program to create opportunities for PA organized by the community?	Have you participated in sporting events, health promotion events, virtual/online activities, and/or other types of community-organized recreational activities?

The questions to assess policy implementation and public participation were selected from the most relevant questions of SPA2021 and TRC2021 that correspond to ISPAH’s guidelines [26, 27]. Test-and retest methods were conducted to test the validity of SPA questions. Detailed methods and the results of validity test has been stated in the previous publication employing the same survey dataset [6]. TRC questions were developed from Global Matrix 3.0 benchmarks indicators [28] that were agreed by 49 countries. Global Matrix itself is a set of common indicators to evaluate PA of a country, developed by Active Healthy Kids Global Alliance. The working group is comprised of researchers, health professionals, and policy makers who focus on physical activity promotion for children and youth worldwide.

### Public participation in PA programs

Similar to the policy implementation, data for evaluation of public participation in PA programs were employed from: 1) TRC2021 for the whole-of-school domain, with children and adolescents (aged 5–17 years, *N* = 6,078) as the eligible respondents; 2) SPA2021 for the workplace domain, with working aged Thais (aged 20–59 years, *N* = 5,895) as the sample; and 3) SPA2021 for the general public which comprised of Thais aged 5 years and over (*N* = 7,847) for the rest of the domains. Questions to assess public participation in each domain are shown in Table 1. Public participation in PA programs was calculated from the percentage of respondents participating in each domain of investment divided by the total number of eligible community members.

### Covariates

The covariates included in the model comprised of gender (male, female), age (5–17, 18 – 59, 60 or over), marital status (single, married, widowed, divorced/separated), educational attainment (primary or less, secondary, post-secondary), occupation (unemployed, full-time student, agriculture, self-employed, work in the formal sector, work in the non-formal sector). Income was grouped into no income, < 3,500 THB (< 100 USD), 3,500–10,000 THB (100–288 USD), 10,001–15,000 THB (288–433 USD), 15,001–30,000 THB (433–866 USD), or > 30,000 THB (> 866 USD). Health status inquired whether the respondents has NCD or not. Area of residence was grouped into urban or rural, and region was categorized into North, Northeast, Central, South, or Bangkok. 

### Grading assignments

In the final stage, we developed a grading scheme (*A to F*) and adopting the Global Matrix 3.0 [28] as overall investment evaluation. The grades were calculated from the average percentage of policy availability, policy implementation, and public participation for each domain in ISPAH’s 8-investments. The purpose of grading was to facilitate a comparison of the different investments and ranking which policy has been more or less readily available, implemented, and accessible (being used) for the public.

### Data analysis

Descriptive statistics were employed to assess the percentage of policy availability, policy implementation, and public participation. Binary logistic regression analysis was used to test statistical associations between explanatory factors and public participation in PA program for each investment domain. We included all covariates to examine how population characteristics associated with their participation across the different domains of investment.

We used IBM SPSS Statistics version 24 for the data analysis. We applied the concept of Maximum Likelihood Estimation (MLE) [29] for binary logistic regression and examined the required basic assumptions and data. We found no multicollinearity between predictor/explanatory variables, no linear relationships between independent variables to log odds, and no extreme outliers. We included all explanatory variables at once as the full model and assigned 0.05 to determine the significance of one variable to public participation in PA program in 8 domains as the dependent variables.

## Results

### Overall investment score and grades

All 384 policy and strategy documents, rules, regulations, legislation, and guidelines on PA collected were successfully reviewed and evaluated. A total of 47 policies were excluded from the analysis because there was no consensus reached by the experts in determining the scores. Overall, policies related to 8-investments on PA obtained a score of 69.8% for its availability, indicating the majority of policies have met the three criteria of CAPPA framework. Among the available policies, 51.8% policies have been implemented at the local or national level, with an average of 38.7% in the public participation rate.

Of the total 337 policies/investment being assessed using CAPPA framework, public education and mass media received the highest availability score (95.1%) followed by sports/recreation for all (85.5%). This means that there have been adequate amounts of policies/programs related to both domains, available either at the national or community level, in various policy stages (agenda setting, formulation, endorsement/legitimization, implementation, evaluation, maintenance or termination), and in any format (formal written, unwritten). Community-wide initiative investments scored lower (62.7%) than whole-of-school, active transport, and active urban design (73.8%). The workplace and healthcare domains received the lowest score, (48.1 and 46.2%, respectively), reflecting a lack of investment in both sectors (Table 2).

**Table 2:** Investment score and grade

**8-investments**	**Investment scores**			**Average score** (%)	**Grades**
	**Policy availability** (%)	**Policy implementation** (%)	**Public participation** (%)		
Whole-of-school approach	73.5	11.6	26.9	37.3	D +
Active transport	73.2	66.3	51.7	63.7	B-
Active urban design	73.8	72.3	52.1	66.1	B-
Healthcare	46.2	77.3	49	57.5	C +
Public education/mass media	95.1	62.4	46.3	67.9	B
Sports/recreation for all	85.5	39.4	51.1	58.7	C +
Workplace	48.1	25.9	23.7	32.6	D
Community-wide initiative	62.7	59.2	8.7	43.5	C-
Average policy score	69.8	51.8	38.7		
Policy adoption rate		74.3^a^	55.5^a^		
Successful implementation			74.7^b^		

A + : 94 – 100%; A: 87 – 93% (highly successful in promoting PA); A-: 80 – 86%; B + : 74 – 79%; B: 67 – 73% (predominantly successful in promoting PA); B-: 60 – 66%; C + : 54 – 59%; C: 47 – 53% (moderately successful in promoting PA); C-: 40 – 46%; D + : 34 – 39%; D: 27 – 33% (less than moderately successful in promoting PA); D-: 20 – 26%; F: under 20% (minimally successful in promoting PA); a) Policy adoption rate: percentage of policies being implemented among the available ones; b) Successful implementation: percentage of public participation in the implemented policies

We explored the gaps between policy availability and implementation in order to provide recommendations for the country in planning and refining PA investment, and to ensure equal opportunity for the Thai population in accessing the investments. We found that, although public education and mass media ranked the first in the score of policy availability, only 62.4% of those policies are being implemented. The gap is even higher in the sports/recreational domain where only 39.4% of the available policies are being implemented. The highest score in the policy implementation was found in healthcare (77.3%) and active urban design (72.3%), whereas the lowest score was for the whole-of-school approach (11.6%). In terms of public participation, this study found that members of the Thai public mostly participated in active urban design, active transport, and sports/recreation domains (52.1, 51.7%, and 51.1%, respectively). Only 8.7% of the sample reported participating in community-wide initiatives (Table 2).

The average score of policy availability, implementation, and public participation in each 8-investment domain is shown in Table 2. Public education and mass media received the highest score (67.9%, grade B), followed by active urban design (66.1%, grade B-) and active transport (63.7%, grade B-) investments. It is noteworthy that the whole-of-school approach and workplace domains received the lowest scores (37.2% and 32.6%, grade D and D + , respectively), suggesting many available policies are not being well-implemented and/or being underutilized by the public.

### Differentials in PA participation across the 8-investment domains

The researchers further examined factors associated with the level of participation in each domain. The whole-of-school programs (IV1) were implemented in primary and secondary schools and, thus, the users of this investment are limited to youth aged 5–17 years. This study found that only 26.9% of youth had access to or participated in the PA program. Girls (OR 0.670, *p*-value 0.000) and older adolescents in secondary schools (OR 0.654, *p*-value 0.000) were less likely to participate in the whole-of-school domain compared to boys and primary school students. Compared to Bangkok residents, those who resided in the south (OR 1.744, *p*-value 0.000), north (OR 1.496, p-value 0.000), and northeast (OR 1.450, *p*-value 0.000) regions of Thailand were more likely to have better access to the whole-of-school investment. There was only a slight difference in the average PA participation rate among those with and without a non-communicable disease (NCD) (38.2 and 38.9%, respectively) (Table 3).

**Table 3:** Probability of public participation across the 8-investment domains

Sociodemographic characteristics	**IV1**					**IV2**					**IV3**					**IV4**				
	**Whole-of-school**					**Active transport**					**Active urban design**					**Health care**				
	PR (%)	OR	95% CI		*p*-value	PR (%)	OR	95% CI		*p*-value	PR (%)	OR	95% CI		*p*-value	PR (%)	OR	95% CI		*p*-value
			Lower	Upper				Lower	Upper				Lower	Upper				Lower	Upper	
*Gender*																				
Male (Ref.)	31.1					54.6					55.1					48.5				
Female	22.8	0.67	0.603	0.744	0.000	49.0	0.839	0.765	0.920	0.000	49.2	0.839	0.764	0.922	0.000	49.4	1.062	0.968	1.165	0.204
*Age group (years)*																				
5–17	26.9					52.9	1.137	0.909	1.422	0.262	58.1	1.278	1.018	1.605	0.034	39.4	0.747	0.597	0.935	0.011
18 – 59 (Ref.)	NA					54.1					55.1					48.4				
60 or over	NA					39.1	0.673	0.569	0.795	0.000	31.7	0.545	0.459	0.647	0.000	62.0	1.603	1.356	1.896	0.000
*Marital status*																				
Single	26.9					53.3	0.997	0.896	1.109	0.953	57.4	1.168	1.049	1.302	0.005	45.9	1.019	0.915	1.134	0.734
Married (Ref.)	NA					51.9					48.6					53.1				
Widowed	NA					41.2	1.020	0.809	1.286	0.866	39.6	1.143	0.900	1.453	0.273	60.8	1.165	0.924	1.468	0.196
Divorced/separated	NA					51.3	0.997	0.802	1.238	0.977	48.7	1.018	0.818	1.268	0.872	48.4	0.914	0.737	1.133	0.411
No response	NA					47.4					46.8					39.7				
*Education*																				
Primary or less (Ref.)	31.1					44.4					43.3					50.1				
Secondary	21.5	0.654	0.585	0.732	0.000	55.0	1.363	1.193	1.559	0.000	53.4	1.202	1.050	1.377	0.008	49.4	1.156	1.011	1.323	0.034
Post-secondary	NA					54.2	1.321	1.136	1.537	0.000	57.2	1.364	1.171	1.590	0.000	48.2	0.989	0.850	1.151	0.891
No response	NA					40.8					40.8					46.6				
*Occupation*																				
Unemployed (Ref.)	NA					43.5					39.8					52.2				
Full-time student	26.9					52.9	1.130	0.918	1.390	0.250	59.5	1.469	1.190	1.813	0.000	41.6	1.136	0.922	1.401	0.232
Agriculture	NA					52.2	1.374	1.116	1.691	0.003	43.1	1.085	0.877	1.342	0.451	59.0	1.202	0.976	1.481	0.083
Self-employed	NA					54.9	1.393	1.161	1.671	0.000	54.1	1.312	1.091	1.578	0.004	48.6	1.012	0.844	1.214	0.895
Work in the formal sector	NA					54.3	1.339	1.127	1.591	0.001	57.3	1.379	1.158	1.643	0.000	50.2	1.197	1.007	1.422	0.041
Work in the non-formal sector	NA					52.6	1.333	1.120	1.588	0.001	50.8	1.246	1.044	1.487	0.015	48.4	1.024	0.860	1.219	0.794
No response	NA					36.8					37.9					42.6				
*Monthly Income (baht)*																				
No income	26.9					51.7	1.034	0.847	1.262	0.743	51.2	0.766	0.625	0.938	0.010	41.8	0.739	0.606	0.902	0.003
< 3,500 THB (< 100 USD)	NA					43.7	0.767	0.635	0.926	0.006	43.6	0.740	0.610	0.897	0.002	53.0	0.893	0.739	1.078	0.239
3,500–10,000 THB (100–288 USD)	NA					51.6	0.863	0.742	1.003	0.055	50.6	0.869	0.746	1.013	0.072	50.6	0.860	0.740	0.999	0.049
10,001–15,000 THB (288–433 USD)	NA					53.3	0.886	0.755	1.040	0.138	52.9	0.869	0.739	1.021	0.089	50.2	0.915	0.780	1.073	0.276
15,001–30,000 THB (433–866 USD)	NA					51.6	0.841	0.718	0.985	0.032	55.7	0.943	0.803	1.107	0.472	46.6	0.822	0.702	0.963	0.015
> 30,000 THB (> 866 USD) (Ref.)	NA					58.5					57.5					51.1				
No response	NA					51.6					55.3					54.4				
*NCD*																				
No NCD	26.8					52.2	0.910	0.822	1.008	0.072	54.3	1.033	0.932	1.146	0.535	45.5	0.719	0.649	0.796	0.000
Has an NCD (Ref.)	27.9					50.5					47.2					56.5				
*Area of residence*																				
Urban	24.1	1.310	1.169	1.469	0.000	51.5	1.080	0.977	1.194	0.131	54.6	1.469	1.327	1.626	0.000	48.3	1.019	0.922	1.127	0.708
Rural (Ref.)	32.1					52.0					47.3					50.3				
*Region*																				
North	30.3	1.496	1.217	1.838	0.000	55.5	1.723	1.471	2.020	0.000	54.4	1.970	1.678	2.313	0.000	52.8	1.635	1.394	1.918	0.000
Northeast	33.7	1.450	1.180	1.780	0.000	56.4	1.738	1.450	2.084	0.000	54.9	1.949	1.623	2.342	0.000	52.0	1.636	1.364	1.963	0.000
Central	16.9	0.614	0.504	0.749	0.000	46.9	1.166	1.002	1.357	0.047	48.3	1.403	1.203	1.635	0.000	45.8	1.226	1.052	1.429	0.009
South	32.8	1.744	1.420	2.142	0.000	57.7	1.844	1.539	2.208	0.000	59.9	2.341	1.948	2.814	0.000	53.7	1.753	1.463	2.101	0.000
Bangkok (Ref.)	24.9					44.3					45.3					40.6				
Constant		0.923			0.565		0.621			0.001		0.402			0.000		0.793			0.095
Sociodemographic characteristics	**IV5**					**IV6**					**IV7**					**IV8**				
	**Public education**					**Sport and recreation for all**					**Workplace**					**Community initiative**				
	PR (%)	OR	95% CI		*p*-value	PR (%)	OR	95% CI		*p*-value	PR (%)	OR	95% CI		*p*-value	PR (%)	OR	95% CI		*p*-value
			Lower	Upper				Lower	Upper				Lower	Upper				Lower	Upper	
*Gender*																				
Male (Ref.)	46.3					54.9					28.4					10.2				
Female	46.4	1.026	0.936	1.125	0.589	47.6	0.778	0.709	0.854	0.000	19.2	0.677	0.598	0.768	0.000	7.2	0.759	0.644	0.894	0.001
*Age group (years)*																				
5–17	40.3	0.795	0.634	0.996	0.046	58.1	1.557	1.242	1.951	0.000	24.1	2.727	2.101	3.540	0.000	6.9	0.576	0.388	0.854	0.006
18 – 59 (Ref.)	47.5					53.6					25.0					9.9				
60 or over	47.5	1.246	1.056	1.471	0.009	32.1	0.610	0.514	0.724	0.000	8.3	0.311	0.213	0.454	0.000	4.9	0.835	0.595	1.171	0.296
*Marital status*																				
Single	45.8	0.971	0.873	1.081	0.593	55.6	1.078	0.968	1.200	0.170	28.9	1.804	1.567	2.078	0.000	10.3	1.384	1.146	1.670	0.001
Married (Ref.)	48.3					48.2					18.3					7.8				
Widowed	44.8	0.918	0.732	1.151	0.460	39.2	1.293	1.020	1.640	0.034	12.2	0.830	0.490	1.406	0.488	4.6	0.971	0.581	1.623	0.909
Divorced/separated	46.9	0.971	0.783	1.203	0.787	49.2	1.032	0.830	1.283	0.778	24.3	1.638	1.224	2.192	0.001	8.6	1.193	0.811	1.755	0.370
No response	39.9					45.3					14.7					5.2				
*Education*																				
Primary or less (Ref.)	40.5					41.4					14.5					5.1				
Secondary	48.8	1.570	1.372	1.796	0.000	54.2	1.557	1.359	1.783	0.000	24.8	1.878	1.531	2.303	0.000	9.5	1.654	1.270	2.154	0.000
Post-secondary	48.9	1.569	1.348	1.827	0.000	56.0	1.745	1.497	2.033	0.000	26.5	2.130	1.691	2.682	0.000	10.4	1.635	1.222	2.188	0.001
No response	32.8					31.7					24.1					4.2				
*Occupation*																				
Unemployed (Ref.)	46.1					38.7					NA					5.0				
Full-time student	44.4	1.317	1.068	1.626	0.010	58.1	1.375	1.116	1.694	0.003	26.9					9.1	1.923	1.323	2.795	0.001
Agriculture	49.8	0.992	0.809	1.218	0.942	42.8	1.178	0.953	1.456	0.131	NA					10.1	1.603	1.095	2.346	0.015
Self-employed	45	0.838	0.699	1.004	0.055	52.0	1.411	1.174	1.696	0.000	14.5					7.3	1.060	0.737	1.523	0.755
Work in the formal sector	49.7	1.039	0.875	1.233	0.663	56.9	1.555	1.306	1.851	0.000	30.2					10.8	1.512	1.090	2.099	0.013
Work in the non-formal sector	43.4	0.804	0.676	0.958	0.014	50.5	1.395	1.169	1.665	0.000	17.2					9.0	1.454	1.037	2.040	0.030
No response	39.5					34.7					NA					6.3				
*Monthly Income*																				
No income	40.6	0.970	0.796	1.183	0.767	52.0	1.094	0.894	1.340	0.382	23.5	0.740	0.561	0.977	0.034	7.2	0.959	0.666	1.379	0.820
< 3,500 THB (< 100 USD)	46.5	1.192	0.988	1.438	0.066	44.2	1.035	0.854	1.254	0.724	20.4	0.683	0.514	0.909	0.009	6.5	0.803	0.565	1.141	0.221
3,500–10,000 THB (100–288 USD)	50.0	1.338	1.151	1.554	0.000	49.5	1.049	0.901	1.220	0.539	19.2	0.750	0.609	0.923	0.007	8.2	0.848	0.651	1.104	0.221
10,001–15,000 THB (288–433 USD)	49.7	1.296	1.106	1.520	0.001	52.4	1.023	0.871	1.201	0.779	26.9	1.044	0.854	1.277	0.676	10.4	1.108	0.849	1.446	0.451
15,001–30,000 THB (433–866 USD)	47.3	1.134	0.969	1.327	0.117	55.9	1.115	0.951	1.307	0.178	25.0	0.873	0.717	1.064	0.178	1.02	1.098	0.842	1.430	0.491
> 30,000 THB (> 866 USD) (Ref.)	50.4					58.5					28.2					10.7				
No response	33.6					40.9					21.9					7.3				
*NCD*																				
No NCD	45.3	0.873	0.788	0.966	0.009	52.6	0.948	0.855	1.051	0.309	25.0	1.275	1.103	1.475	0.001	9.3	1.110	0.923	1.336	0.268
Has an NCD (Ref.)	48.5					47.8					20.1					7.3				
*Area of residence*																				
Urban	45.5	0.951	0.861	1.051	0.322	51.5	1.085	0.981	1.201	0.113	24.0	1.287	1.122	1.475	0.000	8.1	0.916	0.772	1.088	0.317
Rural (Ref.)	47.9					50.3					23.0					9.8				
*Region*																				
North	48.2	1.320	1.127	1.547	0.001	53.3	1.490	1.270	1.749	0.000	27.8	2.242	1.799	2.794	0.000	10.9	2.701	1.949	3.743	0.000
Northeast	49.9	1.409	1.176	1.689	0.000	52.3	1.378	1.148	1.654	0.001	28.1	2.084	1.626	2.671	0.000	11.8	2.840	1.998	4.036	0.000
Central	44.7	1.138	0.977	1.326	0.096	48.5	1.153	0.990	1.343	0.068	21.1	1.420	1.146	1.761	0.001	6.2	1.373	0.984	1.918	0.063
South	47.4	1.298	1.084	1.553	0.005	55.3	1.577	1.315	1.890	0.000	25.0	1.865	1.453	2.394	0.000	10.4	2.477	1.736	3.535	0.000
Bangkok (Ref.)	41.5					47.2					17.4					4.9				
Constant		0.507			0.000		0.421			0.000		0.028			0.000		0.024			0.000

*PR* participation rate; non-response was excluded from the multivariate analysis

For the rest of the investment domains (IV2-IV8), sociodemographic characteristics were found to be associated with participation rate. Sex was significantly associated with participation rate in active transport, active urban design, sports/recreation for all, workplace, and community-wide initiatives. Females were less likely to participate in those domains compared to their male counterparts, but there was no significant association between sex and participation rate in the healthcare and public education/mass media campaign investments. Age was also a consistent predictor of utilization in IV2-8. Compared to the working-aged group, older adults were more likely to use health care facilities (OR 1.603, *p*-value 0.000) and more likely to have been exposed to public education/mass media (OR 1.246, *p*-value 0.009), but were less likely to utilize active transport, active urban design, sports/recreation for all, and workplace investments. Children and adolescents, on the other hand, were more likely to participate in the active urban design (OR 1.278, *p*-value 0.034), sports/recreation for all (OR 1.557, p-value 0.000), and workplace (OR 2.727, *p*-value 0.000) programs, and less likely to use health care, public education, and community-wide initiatives. Marital status served as a less consistent predictor of participation in seven domains of investment. Being single meant that one was 1.8 times more likely to use the workplace facilities (*p*-value 0.000) and 1.168 times more likely (*p*-value 0.005) to participate in active urban design-related initiatives. By contrast, being a widow(er) meant that one was 1.293 times more likely (*p*-value 0.034) to use sports/recreation for all amenities. The probability of using workplace facilities/program was also 1.638 times higher (*p*-value 0.001) among divorced individuals (Table 3).

Socioeconomic status was associated with the level of the sample population’s participation in PA investments. Educational attainment had a strong consistent positive association with the participation rate in the seven investments. Those who attained secondary or post-secondary education had a higher probability to use all the PA programs/investments compared to individuals who attained primary education or less. Likewise, occupation was also positively associated with participation rate in most of the domains. Active transport users were likely to be persons with various full-time occupations (except for students), whereas active urban design amenities were utilized by those who were employed in the formal- or informal sector, self-employed (except those working in agriculture), or were full-time students. Health care investments interestingly, was only significantly associated with formal-sector employees (OR 1.197, *p*-value 0.041). Public education/mass media was more likely to be used by full-time students (OR 1.317, *p*-value 0.010), and less by those in the informal employment sector (OR 0.804, *p*-value 0.014). Full-time students, the self-employed, those who worked in the formal- and informal sectors also had a higher likelihood of using sports/recreational facilities than their unemployed counterparts. Being the least utilized, participation in community-wide initiatives was also associated with occupation. Being a full-time student, working in agriculture, or employed in the formal or informal sector meant that one had a higher likelihood of using community-wide initiatives (Table 3).

Unlike education and occupation, income was a less significant predictor of public participation across the seven domains. Indeed, income had no significant association with utilization of sports/recreation and community-wide initiative investments, but partially contributed to utilization of the other five domains. Compared to those with > 30,000 Thai baht income per month, those with no income were less likely to participate in active urban design, healthcare, and workplace investments. Individuals who earned < 3,500 Thai baht per month were less likely to use active transport, active urban design, healthcare, and workplace investments than their wealthier counterparts. The lower middle-income group (earning 3,500–10,000 Thai baht/month) were 1.338 times more likely (OR 0.000) to use public education investments and were less likely to participate in healthcare and workplace programs than the highest earners (Table 3).

Having one or more debilitating chronic disease was only associated with participation in healthcare, public education, and the workplace. Individuals with no disease were less likely to participate in healthcare and public education/mass media investments but were 1.275 times more likely to use workplace facilities (OR 0.001).

Public participation was also significantly associated with area of residence (urban/rural, region). Living in an urban area was only significantly associated with participation in active urban design (OR 1.469, *p*-value 0.000) and workplace investments (OR 1.287, *p*-value 0.000), while region of residence was associated with overall participation across the seven domains (IV2-8). Compared to those who lived in the capital region (Bangkok), individuals who resided in south, north, and northeast were more likely to participate in all seven investments, whereas those who lived in the central region were more likely to participate in only four domains (active transport, active urban design, healthcare, workplace).

## Discussion

Thailand has been working on health promotion policies for quite some time. This includes its signing of the 2010 Toronto Charter, and through the leadership of the Thai Health Promotion Foundation (ThaiHealth), which is an agency that facilitates cooperation with partners to carry out its missions. One of these missions is to support and promote PA for Thais in order to achieve and maintain good health. PA has also been entered as a national agenda under the country’s Promotion Plan for 2018–30, with an interim plan for the period of 2018–20.

The results of this study found that, of 337 policies across the eight investment domains, 69.8% were readily available, and 51.8% were implemented either at the local or national level, with an average of 38. 7% public participation rate. Public education and mass media received the highest grade (B) among domains. The grading highlighted the comparative levels of policy availability, implementation and public participation, but also revealed gaps in between the three indices. Although the score for public education and mass media is remarkably high in terms of availability (95.1%), only 62.4% of policies were implemented, and less than half the sample 46.3% said they had seen information from the PA campaigns and practiced more health behavior following the promotional messages. The gaps between policy availability with implementation and public participation indicate inequality of access to information on PA, and in the proportion of the sample who said they could apply various strategies to practice PA safely at home and in the community. As the public education delivered through mass media were intended for the general public, children and adolescents were less likely to be exposed to the content, or less interested in following the recommendations in the messages.

‘Workplace’ received the lowest grade among the eight investments (score 32.6%, grade D), indicating that there is a lack of investment in both the formal and informal labor sectors in terms of policy availability, implementation, and public participation. Workplace PA has not been a priority in Thailand, as reflected by the low policy score in the implementation aspect. A previous study found that there were some efforts by employers to provide afterhours sports and recreational PA at the workplace however, that was usually targeted to those workers whose jobs required sedentary behavior [30]. Although it has been acknowledged that PA may boost productivity and improves mental health outcomes [31–33], there has been limited investment in this area. In the absence of national workplace policies/programs, participation in the workplace PA is often constrained by the lack of an enabling organizational structure and policies [33, 34], and lack of a supportive interpersonal climate [35] to allow dissemination of information on the benefits of PA and improve motivation of the employees [36]. Studies have found that successful workplace PA programs have a business plan and rationale for interventions that generate outcomes, including increasing productivity [37–39].

In is noteworthy that the whole-of-school investment scored the second lowest (37.3%, grade D). Although up to 73.5% of PA intervention and policies on whole-of-school were found, only 11.6% were found to be implemented by schools, and only 26.9% of children and adolescents in schools have access to and benefit from the whole-of-school investment. The participation rate was lower among girls, older children and those who resided in an urban area. It should be noted, however, that PA of Thai children and adolescents has been very low for many years. The TRC on PA of children and adolescents found that, on average, only 1 in 4 youth had sufficient MVPA in 2016–22 [40–42]. Furthermore, the ‘whole-of-school’ as a comprehensive approach in PA promotion at school has not been widely implemented in Thailand. A pilot study adopting the whole-of-school approach was conducted in 2017–19 in 14 schools, and more than 400 students were randomly assigned into experimental and control groups. The results of that study found that students who received the intervention (4PC model: Active Policy, Active Program, Active People, Active Place, Active Classroom) demonstrated an increased level of PA and a higher level of happiness at school [12]. At the time of this report, the 4PC model is being scaled up to be implemented nationwide, and the researchers expect to observe a positive impact of the investment in narrowing the inequality gaps in PA participation, particularly among girls, adolescents, and urban youth in the near future.

This study found that only 8.7% of community-wide initiative investments were utilized by the public. The low participation in the program indicates that the existing investments were unable to serve the needs and preferences of their intended beneficiaries. Ideally, community-wide investments would involve all community members in the planning and implementation of the initiatives to ensure sustainability and produce a long-term benefit for all community members [43]. Studies have found that community-based initiatives that are implemented at multiple levels and across multiple sectors are more effective in improving participation and health behavior outcomes. This approach typically includes modifying the environment, creating a supportive policy, engaging community members in all stages of development, and designing programs/interventions that are tailored to the community priorities [44–46]. However, during the desk review, the researchers could not identify whether the actual community initiatives process was in place during planning and implementation. The policies/investments are supposed to narrow the gaps of environmental and structural inequality [47]. However, this study found that the users of the investments were mostly male, young adults (including full-time students, and those employed in agriculture, or the formal/informal sectors), and those with secondary or post-secondary education. Females, children, and adolescents were less likely to participate because the programs did not address their priority interests. There were regional differences in community-wide initiatives participation. For example, residents of the north, south, and northeast regions had higher participation than residents of Bangkok. That finding suggests that participation in the community-wide initiatives was affected by the local government containment measures during the COVID-19 epidemic in Thailand, and where Bangkok and its surroundings were more strictly controlled due to severity of the spread of infection [6].

With an overall score of 57.5% (grade C +), investments in healthcare were utilized by 49% of the sample in the survey. The higher likelihood of utilization among older adults and people with an NCD is not surprising since this group of the population may have a higher concern for their health than the younger and healthy individuals. However, this study also found that only those who worked in formal sector were more likely to use healthcare facilities, whereas those in other occupations were not. Participation/utilization was also low among individuals with lower or medium income. It should be noted that Thailand passed its Social Security Act in 1990 (which covers both public and private sector employees) as well as the Universal Health Coverage Scheme (UCS) (enacted in 2001) which covers any Thai citizen without health insurance from another provider [48]. The low utilization of healthcare investments in PA among the lower- and medium-income individuals (compared to the higher-income) and also among people who worked in the non-formal sector indicates that healthcare seeking behaviors of Thais was determined, in part, by the perceived severity of the disease threat. Typically, the lower-income groups refrain from seeking treatment unless they fall painfully ill. That is because they cannot afford to lose income from leaving work for non-emergencies (and since they are unlikely to be covered by paid sick leave). Obviously, those with higher income have more choices for health care since they can also go to private hospitals and clinics which are located in every province of the country. On the supply side, a previous study found that brief interventions for PA are more likely to be successful and encourage prolonged adherence when it is provided alongside primary health care by trained service providers [49]. That said, the low availability of PA investment in healthcare is largely due to the fact the Thai health system usually administers PA interventions in public hospitals. However, given the unfavorable ratio of health personnel to patients, the PA investment in public hospitals (e.g., brief advice) is often bypassed, and given as part of the prescription service of the pharmacist when dispensing the obligatory take-home medications.

Active urban design and active transport are the most utilized investments in Thailand (both graded B-), followed by sports/recreation for all (grade C +). The patterns of participation and users of the three investments are similar, except that the effect of area of residence (rural/urban) was only significant in the active urban design investment. Females and older adults were less likely to use the three investments, whereas children and adolescents were more likely to participate in PA related to the active urban design and sports/recreation domains. Inequality in PA access has been acknowledged widely, and the existing PA amenities and programs favored the socially-advantaged groups, e.g., males and working-aged adults, rather than for females or older adults [7, 50, 51]. Differential PA participation between Thai males and females also suggests that there is a gender and cultural construct that encourages females to be in a close proximity to their family home or domicile. The traditional gender norms of Thai females may also constrain them from participating in sports at their convenience because of the conflict between their culturally-prescribed domestic duties and PA opportunity (e.g., evening exercise vs preparing dinner) [6]. Individuals with secondary and post-secondary education were more likely to participate in the three investments (than those with only primary education), because of the combined effect of knowledge and opportunity. Those with higher education are likely to be more informed of the benefit of PA, and have more opportunity and access to PA amenities in three domains [52]. The insignificance/inconsistency of income in predicting PA participation in three domains was likely because of the reluctance of Thais in revealing their actual income to avoid taxation. Similar to community-wide initiatives, regional differences in public participation in the three domains was also likely to be affected by the local government measures in containing COVID-19.

This study offers a simple yet comprehensive method to add to the body of knowledge in policy evaluation, particularly in relation to the 8-investments strategy that is recommended to boost PA. Each investment was assessed comprehensively, and evaluated across three different dimensions: Availability, extent of implementation, and accessibility to the public. The use of the CAPPA framework to score policy availability also served as one of the strengths of this study. The data used to evaluate policy implementation and public participation were derived from national samples of the Thai population, thus allowing some generalization of the findings. This comprehensive policy evaluation of ISPAH’s 8-investments took into account WHO’s GAPPA, and the methods should aid countries in aligning the policies and programs to meet the global indicator targets and goals. However, there are some limitations of the study. The evaluation of policy implementation relied on the perceptions of the intended beneficiaries, instead of objective observation of the actual programs. Another limitation is that data collection occurred during the COVID-19 epidemic and, therefore, the findings do not necessarily represent the situation in the absence of epidemic conditions. For the policy resources allocation, identical scores were given for both having management resources and having budget allocated due to limited access to budget documents. It should be noted however, although the documents may not be accessible to the public, there is clear evidence that the budget is available for particular investments. Lastly, behavioral and health outcomes could not be determined due to the cross-sectional nature of the survey.

## Conclusions

Workplace, whole-of-school approach, and community-wide initiatives scored the lowest in this policy evaluation, while public education and mass media ranked the highest. The findings suggest that future policy and programs should focus on the least accessible and least utilized investments because successful investments in the 8 domains will influence PA level of the population. This study showed, with a varying degree of policy availability and accessibility, public participation in PA investments is likely to be constrained by biological and socioeconomic inequality. Future investments should, therefore, expand coverage and inclusivity. Target-specific and tailored programs should be designed so that they are appropriate to the needs and lifestyle of each group of the population, since that should increase accessibility and opportunity. Collective action for PA should be promoted, extending from the policy level to frontlines to ensure equal access and participation of all segments of the population in need.

### Supplementary Information

**Additional file 1:** **Supplementary material.** Summary table for policy availability.

## Data Availability

The datasets used and/or analyses during the current study are available from the corresponding author on reasonable request.

## Abbreviation

PA: Physical activity
MVPA: Moderate-to-vigorous-physical activity
ISPAH: International Society for Physical Activity and Health
GAPPA: Global Action Plan on Physical Activity
WHO: World Health Organization
DPA: Daily Physical Activity
CAPPA: Comprehensive Analysis of Policy on Physical Activity
TRC: Thailand Report Card Survey
SPA: Surveillance on Physical Activity
RoI: Returns of Investment
