# Supplementary material for: Assessing physical activity promotion in different settings and how its associated with public participation during COVID-19 epidemic: evidence from national policy evaluation
Source: BMC Public Health. 2023 Sep 12;23:1775. doi: 10.1186/s12889-023-16690-9 (PMC10496167; doi:10.1186/s12889-023-16690-9)
Supplement: Supplementary file 1 — Additional file 1: Supplementary material. Summary table for policy availability. [file 12889_2023_16690_MOESM1_ESM.pdf]

Supplementary material: Summary table for policy availability.

Domain	n of policy in each domain	Scoring scheme	Score: No consensus	Score: 0	Score: 1	Score: 2	Score: 3	Max score for each domain	Weighted score obtained	Final score for each domain (in percentage)
1 Whole-of-school approach	51	n of policy being scored	2	3	6	18	22	147		73.5
		weighted score		0	6	36	66		108	
2 Active transport	42	n of policy being scored	1	3	2	20	16	123		73.2
		weighted score		0	2	40	48		90	
3 Active urban design	77	n of policy being scored	7	4	11	21	34	210		73.8
		weighted score		0	11	42	102		155	
4 Healthcare	35	n of policy being scored	4	6	12	8	5	93		46.2
		weighted score		0	12	16	15		43	
5 Public education/mass media	60	n of policy being scored	6	0	2	4	48	162		95.1
		weighted score		0	2	8	144		154	
6 Sports/recreation for all	26	n of policy being scored	3	0	3	4	16	69		85.5
		weighted score		0	3	8	48		59	
7 Workplace	38	n of policy being scored	11	3	12	9	3	81		48.1
		weighted score		0	12	18	9		39	
8 Community-wide initiative	55	n of policy being scored	13	1	12	20	9	126		62.7
		weighted score		0	12	40	27		79	
Total policies being evaluated in 8 domains	384		47							

* No consensus reached among experts; policy were excluded from scoring
